# Rapid Progression of Dermatomyositis in an Elderly Patient With Oropharyngeal and Pulmonary Involvement: A Case Report

**DOI:** 10.7759/cureus.84267

**Published:** 2025-05-17

**Authors:** Emily O Broad

**Affiliations:** 1 Emergency Department, St Helens and Knowsley NHS Trust, Liverpool, GBR

**Keywords:** dermatomyositis, electromyography (emg), gottron papules, heliotrope sign, intravenous immunoglobulins (ivig), jo-1 antibodies, ro-52 antibodies

## Abstract

Dermatomyositis is a rare autoimmune condition characterized by proximal muscle weakness and distinctive skin manifestations, with increased age being a significant negative prognostic factor. This case presents a 79-year-old male who presented to the emergency department with a six-week history of fatigue, weight loss, progressive symmetrical muscle weakness, and hallmark rashes, including a heliotrope rash and Gottron papules. Despite discontinuing statin therapy, thought to be a potential contributing factor to his symptoms, his condition quickly worsened, and investigations confirmed dermatomyositis through positive autoantibodies and imaging. Treatment with corticosteroids was initiated, but he continued to deteriorate rapidly, leading to dysphagia, respiratory distress, and aspiration pneumonia, which ultimately resulted in death just 28 days after initial presentation. This case emphasizes the potential for rapid progression of dermatomyositis in elderly patients, particularly those with oropharyngeal and pulmonary involvement, and highlights the importance of early recognition and multidisciplinary management.

## Introduction

Dermatomyositis is a rare, autoimmune inflammatory condition first described by Ernst Leberecht Wagner in 1868 [1], affecting approximately 1.1 per 100,000 individuals annually [2], with increasing age being a significant negative prognostic factor [3]. It is characterized by proximal muscle weakness and hallmark skin rashes, key for diagnosis as per Bohan and Peter criteria [4], including a heliotrope rash (violaceous discoloration around the eyes) and Gottron papules (raised, scaly lesions over bony prominences) [5]. Approximately 28% of cases are associated with malignancy [6]. Other reported triggers include viral infections, silica exposure, and certain medications, such as hydroxyurea, statins, cyclophosphamide, and tumor necrosis factor (TNF) inhibitors [7]. While often a chronic condition, with five-year survival rates ranging between 56% and 81.9% [8], dermatomyositis can sometimes progress rapidly, particularly in elderly patients. Those over 65 years old have significantly higher rates of mortality, reduced chances of remission, and increased risk of complications such as aspiration pneumonia, respiratory distress, and esophageal impairment [9]. Early recognition of key clinical signs and initiation of appropriate treatment, such as corticosteroids or intravenous immunoglobulin (IVIG), are crucial for improving outcomes [10].

## Case presentation

**Figure 1:**
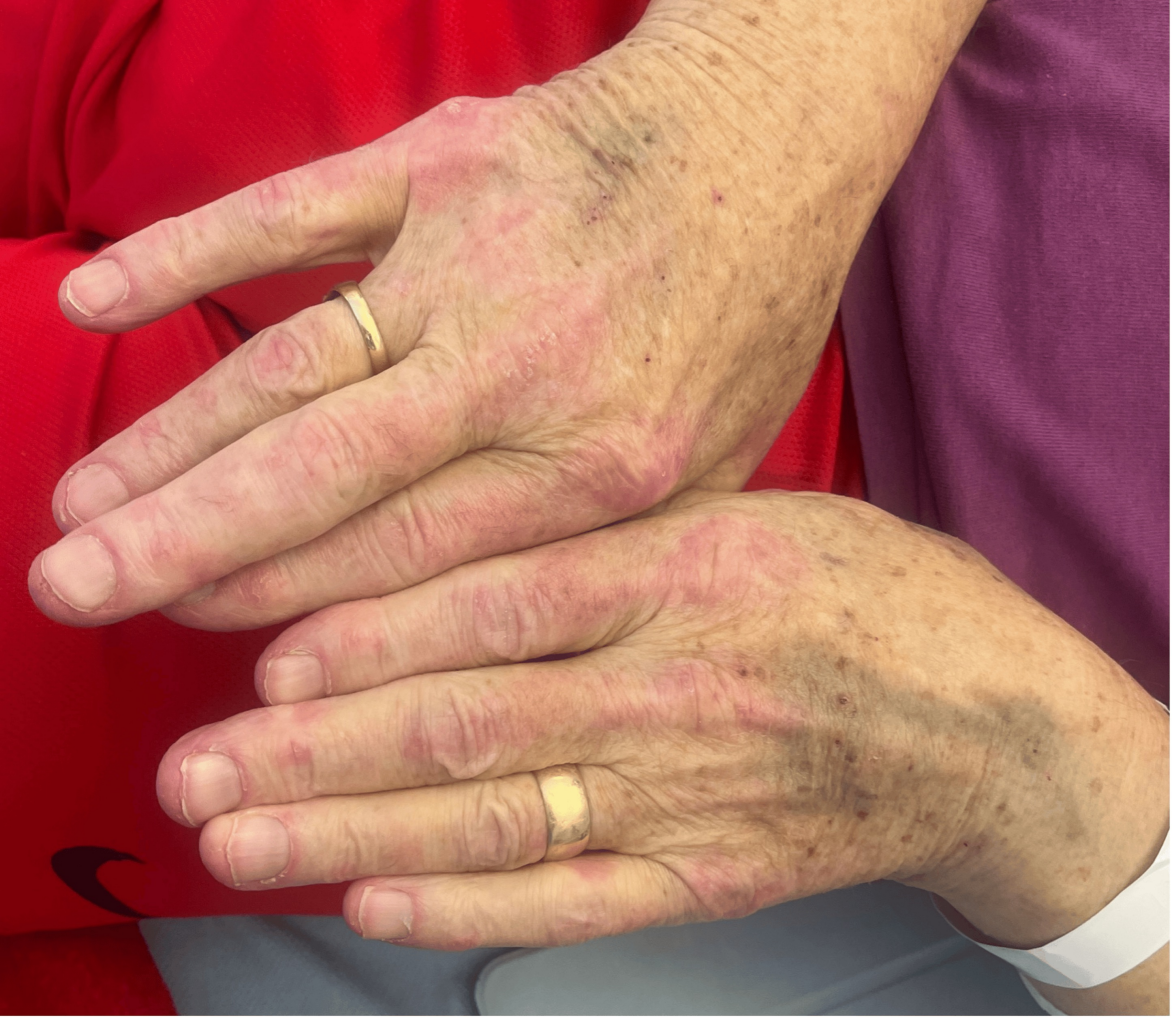
Gottron papules overlying the patient's metacarpophalangeal and interphalangeal joints

A 79-year-old male with a history of previous vocal cord carcinoma, type 2 diabetes, chronic kidney disease, and atrial fibrillation presented to the emergency department with a six-week history of extreme fatigue, weight loss, anorexia, and characteristic skin rashes, including a heliotrope rash, Gottron papules (Figure 1), and a shawl sign. He also reported progressive, symmetrical muscle weakness, significantly impacting his ability to perform daily activities, such as sitting up in bed and reaching for objects.

Initial observations were unconcerning, with a borderline tachycardia. Physical examination revealed proximal muscle weakness (power 4 out of 5 in both upper and lower limbs), and laboratory tests showed elevated inflammatory markers and an elevated creatine kinase (CK) level of 1146 U/L. Given the suspicion that his atorvastatin therapy may be contributing to his symptoms, it was discontinued. While this yielded an improvement in CK levels, to 567 U/L, his condition continued to worsen, raising concerns for an underlying inflammatory myopathy.

**Figure 2:**
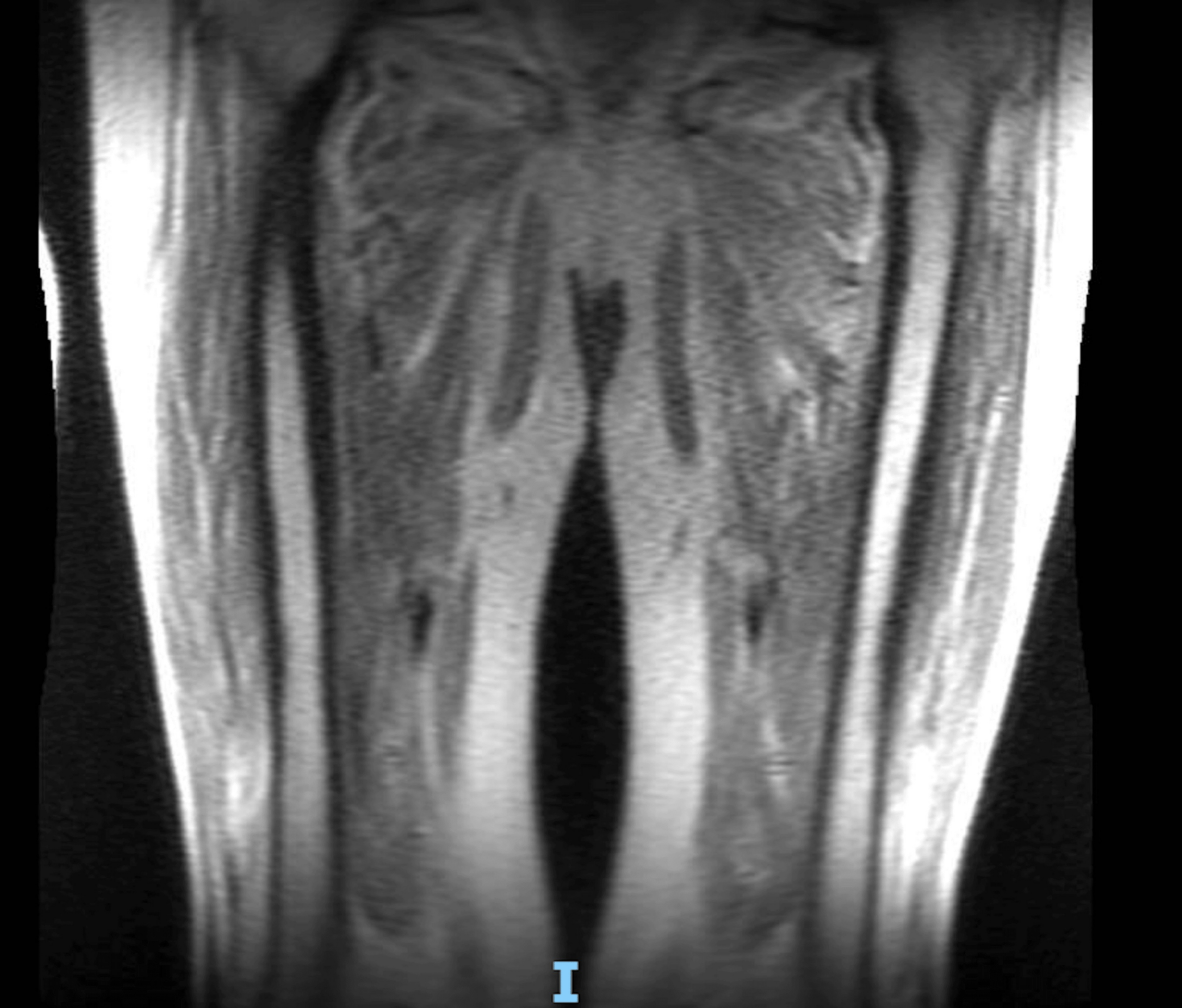
MRI of the femur demonstrating extensive muscle edema in both the thighs

Further investigation revealed positive autoantibodies to Ro-52 (a myositis-associated antibody) and TIF, with weak positivity for Jo-1 (both myositis-specific antibodies), suggesting dermatomyositis. Electromyography (EMG) and magnetic resonance imaging (MRI) of the thigh (Figure 2) later confirmed extensive muscle edema and proximal myopathy, while computed tomography (CT) imaging ruled out an underlying disseminated malignancy. In light of these findings, rheumatology initiated a trial of oral corticosteroids (40 mg prednisolone once daily). However, the patient's condition continued to deteriorate, with new-onset dysphagia requiring nasogastric tube feeding. By day six of oral steroids, his CK levels remained high at 531.

His treatment was therefore escalated to intravenous methylprednisolone and IVIGs. Despite this, he continued to deteriorate, triggering three medical emergency team (MET) calls over the next three days, initially scoring six on the national early warning score (NEWS) with a heart rate of 134, respiratory rate of 29, and a new 15 L oxygen requirement to maintain saturations above 95%. On examination, he had bilateral pulmonary crackles, and treatment for aspiration pneumonia was commenced with intravenous tazocin, as per trust guidelines. Intravenous bisoprolol was also given in an attempt to control his tachycardia. However, his clinical status continued to rapidly decline.

After further consultation with rheumatology, the decision was made to transition to palliative care. The patient passed away just four days after IVIG initiation and 28 days after initial presentation, with aspiration pneumonia as the immediate cause of death and dermatomyositis as the underlying cause.

## Discussion

Dermatomyositis is a rare but potentially devastating autoimmune condition that presents with both muscular and cutaneous features. As evidenced by this case, the disease can vary significantly in its course, with some patients experiencing a rapid and aggressive progression. Literature suggests that while many patients with dermatomyositis respond well to first-line corticosteroids [11], elderly patients may develop a more aggressive form of the disease, requiring further escalation of treatment. A retrospective study of 79 patients demonstrated that those over 65 years old often have a more severe and rapid disease course, with increased frequency of complications like esophageal involvement, aspiration pneumonia, and respiratory failure, and therefore increased overall mortality [12]. This may partly be attributed to immunosenescence, age-related alterations in immune function, which can contribute to disease progression and reduced response to treatment [13]. This case mirrors these findings, emphasizing the challenge of managing dermatomyositis in elderly patients, where rapid disease progression and respiratory involvement may make aggressive treatment ineffective.

This case further highlights the importance of being vigilant for respiratory or esophageal complications in patients with dermatomyositis. The association between dermatomyositis and respiratory diseases, such as interstitial lung disease, pulmonary fibrosis, and aspiration pneumonia, has been well established in the literature, with lung complications sometimes presenting prior to myopathic manifestations [14]. Other studies have also documented the frequent appearance of oropharyngeal muscle involvement in dermatomyositis patients [15,16], emphasizing a role for early swallowing assessments and consideration of nasogastric feeding when indicated.

IVIG represents a key therapeutic option in cases of refractory or rapidly progressive dermatomyositis. A 2010 review of 73 patients reported complete resolution of esophageal dysfunction with IVIG therapy in 82.2% of individuals unresponsive to corticosteroids [17]. A more recent meta-analysis (2023) further supports the efficacy of IVIG, demonstrating improvements in muscle strength and serum creatine kinase levels, alongside an overall favorable safety profile [18]. Notably, the efficacy of IVIG in a large, randomized, placebo-controlled trial (ProDERM) suggests that IVIG may be beneficial as an early, first-line therapy rather than being reserved for steroid-refractory cases [10]. This raises the question of whether earlier initiation of IVIG in this patient, prior to the development of severe complications, might have favorably altered the clinical trajectory. Despite these promising findings, response to IVIG is not universal, particularly in advanced disease or in the presence of poor prognostic indicators such as underlying malignancy or myositis-specific autoantibodies, including anti-Ro52 antibodies as seen in this case [19,20]. This is consistent with the present case, where the patient did not show improvement despite aggressive treatment with both IVIG and methylprednisolone, underscoring the complexity and unpredictability of dermatomyositis in some patients.

## Conclusions

This case highlights the need to consider dermatomyositis as a differential in elderly patients presenting with non-specific features such as progressive weakness, dysphagia, raised CK, or distinctive hallmark skin rashes. Early recognition and prompt multidisciplinary management, including early consideration of IVIG initiation, are crucial to reducing the risk of complications and mortality, particularly given the potential for rapid oropharyngeal and pulmonary involvement in this cohort.
